# Building Block Engineering toward Realizing High-Performance Electrochromic Materials and Glucose Biosensing Platform

**DOI:** 10.3390/bios13070677

**Published:** 2023-06-25

**Authors:** Aliekber Karabag, Dilek Soyler, Yasemin Arslan Udum, Levent Toppare, Gorkem Gunbas, Saniye Soylemez

**Affiliations:** 1 Faculty of Science, Department of Chemistry, Middle East Technical University, Ankara 06800, Turkey; aliekber.karabag@odtugunam.org (A.K.); toppare@metu.edu.tr (L.T.); 2 METU Center for Solar Energy Research and Applications (ODTU-GUNAM), Ankara 06800, Turkey; 3 Faculty of Engineering, Department of Biomedical Engineering, Necmettin Erbakan University, Konya 42090, Turkey; 22822013003@erbakan.edu.tr; 4 Technical Sciences Vocational Schools, Gazi University, Ankara 06500, Turkey; y.udum@gazi.edu.tr; 5 Department of Polymer Science and Technology, Middle East Technical University, Ankara 06800, Turkey; 6 Department of Biotechnology, Middle East Technical University, Ankara 06800, Turkey

**Keywords:** selenophene, 3,4-ethylenedioxythiophene (EDOT), thieno[3,4-*c*]pyrrole-4,6-dione (TPD), conjugated monomers, Stille cross-coupling, optoelectronic and biosensing properties

## Abstract

The molecular engineering of conjugated systems has proven to be an effective method for understanding structure–property relationships toward the advancement of optoelectronic properties and biosensing characteristics. Herein, a series of three thieno[3,4-*c*]pyrrole-4,6-dione (TPD)-based conjugated monomers, modified with electron-rich selenophene, 3,4-ethylenedioxythiophene (EDOT), or both building blocks (**Se-TPD**, **EDOT-TPD**, and **EDOT-Se-TPD**), were synthesized using Stille cross-coupling and electrochemically polymerized, and their electrochromic properties and applications in a glucose biosensing platform were explored. The influence of structural modification on electrochemical, electronic, optical, and biosensing properties was systematically investigated. The results showed that the cyclic voltammograms of EDOT-containing materials displayed a high charge capacity over a wide range of scan rates representing a quick charge propagation, making them appropriate materials for high-performance supercapacitor devices. UV-Vis studies revealed that EDOT-based materials presented wide-range absorptions, and thus low optical band gaps. These two EDOT-modified materials also exhibited superior optical contrasts and fast switching times, and further displayed multi-color properties in their neutral and fully oxidized states, enabling them to be promising materials for constructing advanced electrochromic devices. In the context of biosensing applications, a selenophene-containing polymer showed markedly lower performance, specifically in signal intensity and stability, which was attributed to the improper localization of biomolecules on the polymer surface. Overall, we demonstrated that relatively small changes in the structure had a significant impact on both optoelectronic and biosensing properties for TPD-based donor–acceptor polymers.

## 1. Introduction

Semiconducting π-conjugated materials with well-defined structures have drawn considerable attention owing to their low fabrication cost and ease of processability and tunability of their electrochemical and optical properties through the modification of chemical structures [1,2,3,4,5,6,7,8]. To realize desirable characteristics for commercial applications such as electronic paper [9], smart window [10], solar cells [11,12,13,14,15], electrochromic displays [16,17,18], and medical utilizations [19,20], great efforts have been devoted to designing and synthesizing novel π-conjugated materials via the assembly of available building blocks and adopting wide-ranging functional groups [21,22,23,24,25].

Electrochromism is a distinctive characteristic of certain materials that experience an electrochemical-induced change in coloration [26]. During the last few decades, electrochromic π-conjugated materials have drawn the attention of the biosensing community thanks to their capacity to combine the sensitivity of electrochemical techniques with the intuitive readout of optical sensors [27]. Although still a growing technology, electrochromic-based biosensors are on the rise with various targets (e.g., pesticides, bacteria, cancer biomarkers, and metabolites) being pursued by utilizing electrochromic materials as transducers [28].

In addition to the use of electrochromism itself in biosensing, the utilization of conjugated polymers in electrochemical biosensing platforms has shown strong potential in the last two decades [29,30]. Several substrates have been targeted over the years with glucose sensing leading the pack due to its significance in health monitoring; considering that millions of people worldwide have diabetes, it is important to monitor glucose levels to control the disease. If this condition is not treated in a timely manner, it might cause serious complications such as kidney failure, a stroke, a heart attack, or amputation [31]. In addition, determining the glucose concentration is especially crucial for various food products, mostly because of the Maillard reaction, whereby glucose browns food during drying and long-term storage. Therefore, a crucial measurement in the quality control of food products is the quantitative determination of glucose [32]. Additionally, glucose sensing became the benchmark in biosensing for the development of new sensing platforms due to the availability of several studies over the years.

For the design of conjugated systems in materials and health applications, redox-active, low-cost, and highly stable thiophene has been the go-to building block and has also been integrated into many electron-rich donor units that are mainly implemented in the generation of donor–acceptor-type conjugated polymers. Structurally similar to thiophene, other hetero atom-containing heterocycles, such as furan [33], pyrrole [34], pyridine [35], and selenophene, have also been used extensively. Among the various conjugated building blocks, 3,4-ethylenedioxythiophene (EDOT) is recognized as a material of interest due to its exceptional properties in electronic [36], bioelectronic [37], and optoelectronic [38] applications. Replacing thiophene with EDOT results in lower oxidation potential, higher hydrophilicity, and a lower band gap of its corresponding conjugated polymer [39]. Existing oxygen (O) atoms in its structure play a significant role in stabilizing the positive charges in EDOT-containing polymer chains, and offer opportunities for enhancing the interactions with biological entities [40]. Modifications of sulfur (S) to selenium (Se) generally lead to significant changes in the molecular properties, mainly due to the difference in atomic sizes and polarizability [41,42]. Such building blocks also provide stronger intermolecular interactions to accelerate interchain/intermolecular charge transportation to enhance charge-carrier mobilities [43]. Furthermore, the more electron-rich and polarizable selenophene improves the quinoidal character of the resultant polymers, leading to reduced optical band gaps [44]. Considering π-conjugated systems, the most successful frameworks generally possess electron “push – pull” structures, achieved via the combination of electron-poor acceptor units with electron-rich donor moieties [45]. Among the electron-accepting-type frameworks, the thieno[3,4-*c*]pyrrole-4,6-dione (TPD) scaffold, one of the promising electron-accepting units, has several advantages, mainly providing facile synthesis, great coplanarity, a robust π-π stacking feature, and strong electron transporting, owing to the ease of switching to its quinoid form [46]. In addition to these, this unique symmetric scaffold can be modified not only with numerous functional groups from the reactive sides of the α-positions of the thiophene heterocyclic ring, but also further with *N*-alkylation to gain new functionalities by preserving its remarkable optoelectronic properties [47].

In this paper, a family of TPD-modified conjugated monomers, namely, **Se-TPD**, **EDOT-TPD**, and **EDOT-Se-TPD**, incorporating two different chalcogen-containing heterocycles, selenophene, and EDOT, was initially designed and synthesized utilizing classical Pd-catalyzed Stille cross-coupling. Then, the effects of molecular structure on the electronic structure and the redox properties of these materials were comprehensively explored through cyclic voltammetric and spectroelectrochemical studies. Lastly, their biosensing characteristics were investigated in detail. Electrochemically produced selenophene-containing polymer films have rarely been investigated in the literature [48,49,50,51]. Systematical and comparative studies on the TPD-modified conjugated monomers functionalized by chalcogen-containing heterocycles are essentially missing in the literature, especially regarding their utilization in high-performance biosensor systems. The systematic study revealed that ***poly*(EDOT-TPD)** showed the best performance in both electrochromic and sensing applications in this work. The sensing platform based on this polymer was demonstrated to be effective for glucose sensing in real beverage samples.

## 2. Materials and Methods

All necessary commercial chemicals for the synthetic work in this study were purchased from Sigma-Aldrich and Tokyo Chemical Industry (TCI) Chemicals and used as received. All reactions were conducted under an inert atmosphere, unless otherwise specified. Merck silica gel 60 (particle size: 0.063–0.200 mm, 70–230 mesh ASTM) as the stationary phase was used in column chromatography. Glucose oxidase (GOx, β-D-glucose: oxygen 1-oxidoreductase, EC1.1.3.4, 17,300 units/g solid from *Aspergillus niger*) and β-D-glucose were provided by Sigma-Aldrich. All structural analyses (^1^H and ^13^C) of the isolated products were conducted using a Bruker Spectrospin Avance DPX-400 Spectrometer with the internal reference as trimethylsilane (TMS) in commercial deuterated chloroform-*d* (CDCl_3_), obtained from Sigma-Aldrich. Additional structural analyses were performed using the Waters SYNAPT G1 model of high-resolution mass spectrometry (HRMS). Transparent, conductive indium tin oxide (ITO) coatings on glass slides, required for both electrochemical and optical studies, were bought from Delta Technologies. All spectroelectrochemical studies were performed using GAMRY Reference 600 Potentiostat/Galvanostat and Solarton 1285, with a conventional three-electrode cell configuration in 0.1 M tetrabutylammonium hexafluorophosphate (TBAPF_6_) as the supporting electrolyte in dichloromethane (CH_2_Cl_2_) or acetonitrile (ACN) organic solvent or in a mixture of them. A platinum (Pt) wire, Ag wire, and indium tin oxide (ITO)-coated glass slide were used as the counter electrode, the pseudo reference electrode (+0.3 V vs. Fc/Fc^+^), and the working electrode, correspondingly. Moreover, amperometric measurements were performed using the AUTOLAB PGSTAT 204 Analysis System, supported by a NOVA software package (Metrohm, The Netherlands). Graphite electrodes (Ringsdorff Werke GmbH, Bonn, Germany, type RW001, 3.0 mm diameter with 13% porosity) were used as the working electrode during biosensor studies. All optical studies were carried out using a JASCO V-770 UV-Vis-NIR spectrophotometer. Furthermore, using FEI Quanta 400F models, field emission scanning electron microscopy (FE-SEM) pictures were taken.

### 2.1. General Experimental Procedure of Mono-Stannylation Reaction

The mono-stannylation reactions of molecules **2**-**3** (selenophene and EDOT) were individually performed via Pd-catalyzed Stille cross-coupling according to the literature, with small modifications [52,53]. Initially, molecules **2**-**3** (1.0 eq.) were separately added into the empty Schlenk flask, previously well dried and thoroughly vacuumed, and this was followed by filling it with argon (Ar) gas three times to make the environment fully inert. Then, these starting materials were dissolved in anhydrous toluene (PhMe). Into the solutions, *n*-BuLi solution (1.05 eq., 2.50 M in hexanes) was presented dropwise at −78 °C and this was stirred at this low temperature for 1.5 h. Later, tributyltin chloride (1.05 eq.) was transferred into the highly reactive 2-thienyllithium solution in one portion at −78 °C. The resultant mixture was stirred at room temperature overnight under an Ar atmosphere and dark environment. After that, each reaction was terminated with the addition of H_2_O and this was followed by dilution with Et_2_O. These suspensions were separately extracted with Et_2_O three times. The combined organic layers were dried over MgSO_4_, filtrated, and then evaporated under reduced pressure. The isolated crude products (molecules **4**-**5**) were freshly used for the next step without further purification.

**Figure A1:**
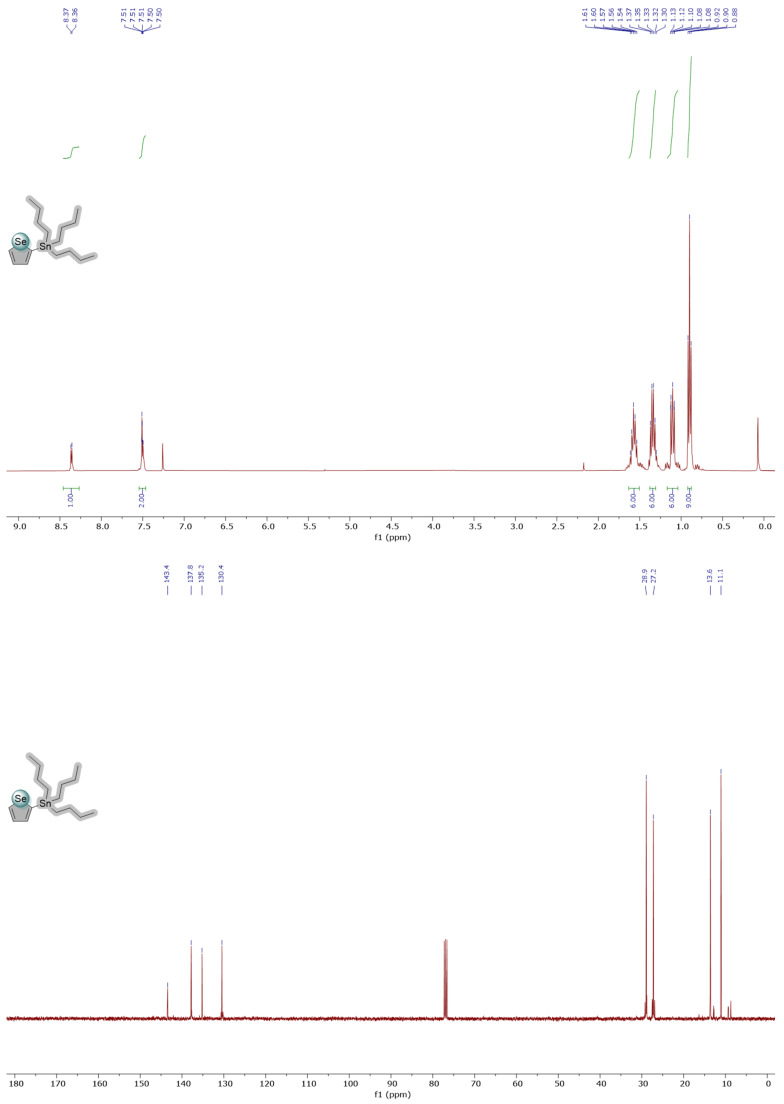
^1^H and ^13^C NMR spectra of molecule **4** in CDCl_3_.

**Molecule 4** (Figure A1). Yield 99%. Yellowish liquid. ^1^H NMR (400 MHz, CDCl_3_): δ 8.36 (d, *J* = 4.8 Hz, 1H), 7.54–7.46 (m, 2H), 1.57 (*p*, *J* = 7.4 Hz, 6H), 1.34 (*p*, *J* = 7.4 Hz, 6H), 1.10 (d, *J* = 7.3 Hz, 6H), 0.90 (t, *J* = 7.3 Hz, 9H); ^13^C NMR (100 MHz, CDCl_3_): δ 143.4, 137.8, 135.2, 130.4, 28.9, 27.2, 13.6, 11.1.

**Figure A2:**
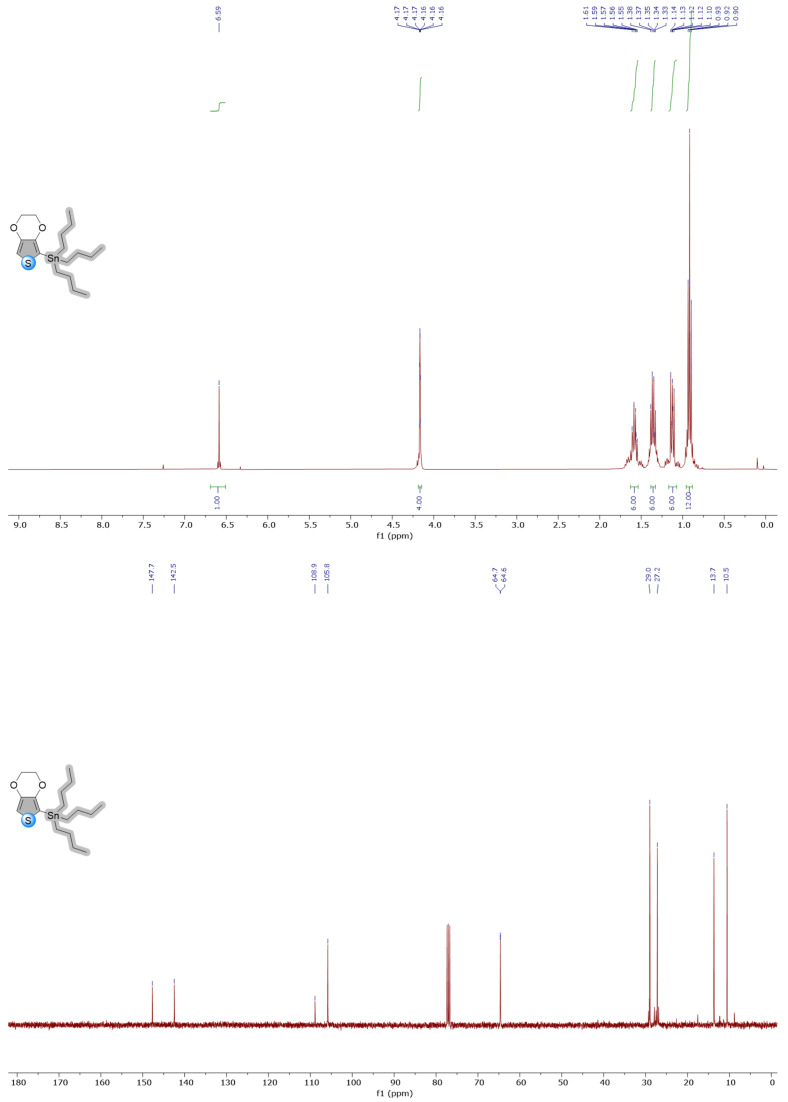
^1^H and ^13^C NMR spectra of molecule **5** in CDCl_3_.

**Molecule 5** (Figure A2). Yield 97%. Yellowish liquid. ^1^H NMR (400 MHz, CDCl_3_): δ 6.59 (s, 1H), 4.18–4.15 (m, 4H), 1.57 (*p*, *J* = 7.2 Hz, 6H), 1.36 (*p*, *J* = 7.3 Hz, 6H), 1.12 (*p*, *J* = 7.3 Hz, 6H), 0.92 (t, *J* = 7.3 Hz, 12H); ^13^C NMR (100 MHz, CDCl_3_): δ 147.7, 142.5, 108.9, 105.8, 64.7, 64.6, 29.0, 27.2, 13.7, 10.5.

### 2.2. General Experimental Procedure of Typical Stille Cross-Coupling

Conventional Stille cross-couplings were effectively performed between previously synthesized mono-stannylated compounds, containing selenophene (SE-Sn(C_4_H_9_)_3_: molecule **4**) and EDOT (EDOT-Sn(C_4_H_9_)_3_: molecule **5**), and bis-brominated TPD central core skeleton with *n*-octyl substituent (Br-TPD(C8)-Br; molecule **1**) according to the literature, with small modifications [54,55]. Molecule **1** (1.0 eq.) was added to the two different empty Schlenk flasks, previously well dried and thoroughly vacuumed, and this was followed by filling them with Ar gas three times to make the environment fully inert. Then, slightly excess amounts of molecules **4-5** (1.1 eq.) were separately transferred into the flasks. These Stille cross-coupling reagents were dissolved in anhydrous PhMe. To get rid of the undesired oxygen in the medium, the reaction mixture was degassed with Ar for at least 30 min. A catalytic amount of Pd(PPh_3_)_4_ was presented into these degassed suspensions and resultant reaction mixtures were stirred at the reflux temperature of PhMe overnight under an Ar atmosphere and dark environment. Each reaction was ended by the evaporation of the reaction solvent under reduced pressure. The remaining residue was purified via silica gel column chromatography using a suitable solvent system. All collected organic phases were checked using thin layer chromatography (TLC) and the corresponding phases were evaporated, including the only product under reduced pressure. Each isolated product was transferred into a well-dried glass vial and stored in an Ar-filled vial for further characterizations (i.e., electrochemical, optical, and biosensing properties). The structural analyses of the desired products were individually performed via NMR spectroscopy (^1^H and ^13^C NMR).

**Figure A3:**
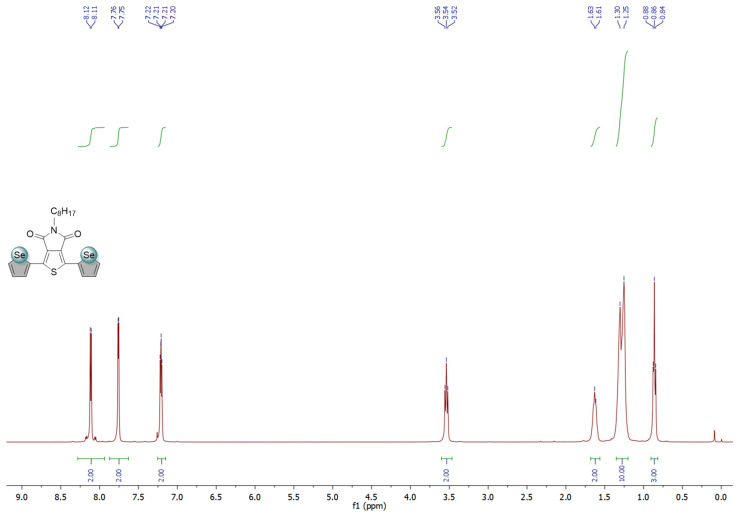

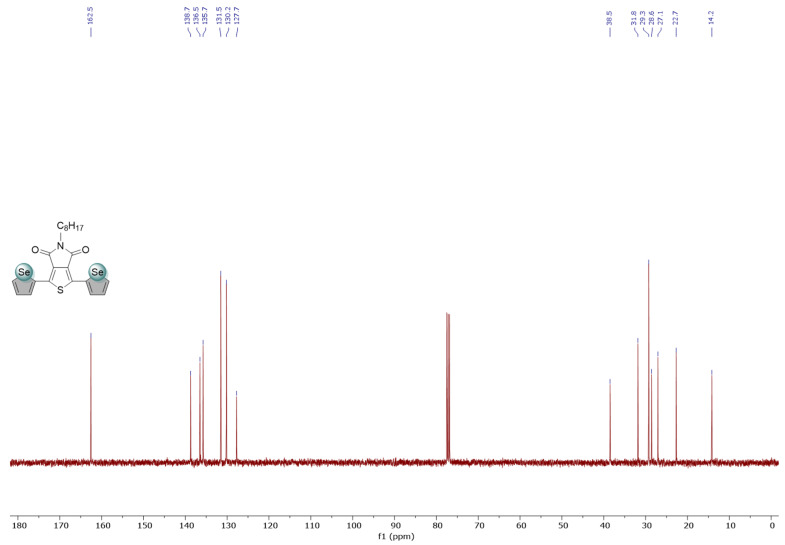
^1^H and ^13^C NMR spectra of **Se-TPD** in CDCl_3_.

**Se-TPD** (Figure A3). Yield 97%. Yellow crystals. ^1^H NMR (400 MHz, CDCl_3_): δ 8.11 (d, *J* = 5.6 Hz, 2H), 7.76 (d, *J* = 3.7 Hz, 2H), 7.21 (dd, *J* = 5.5, 4.0 Hz, 2H), 3.54 (t, *J* = 7.4 Hz, 2H), 1.68–1.56 (m, 2H), 1.28 (d, *J* = 20.5 Hz, 10H), 0.86 (t, *J* = 6.4 Hz, 3H); ^13^C NMR (100 MHz, CDCl_3_): δ 162.5, 138.7, 136.5, 135.7, 131.5, 130.2, 127.7, 38.5, 31.8, 29.3, 28.6, 27.1, 22.7, 14.2.

**Figure A4:**
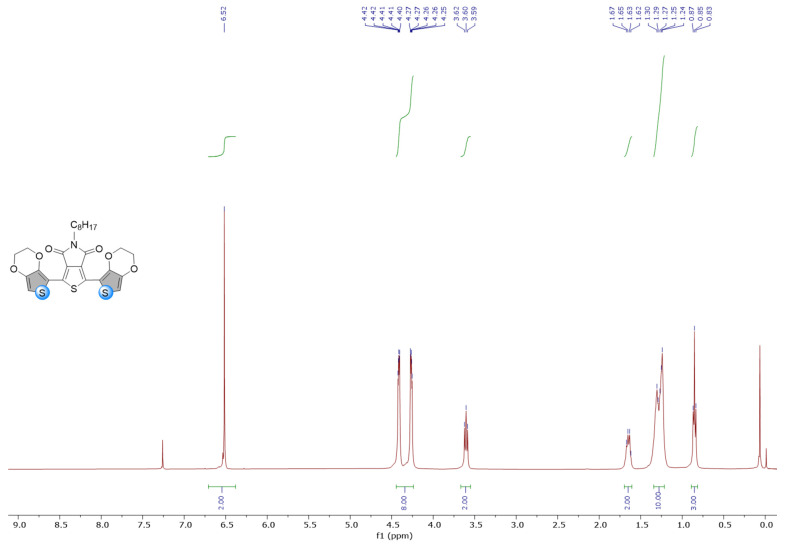

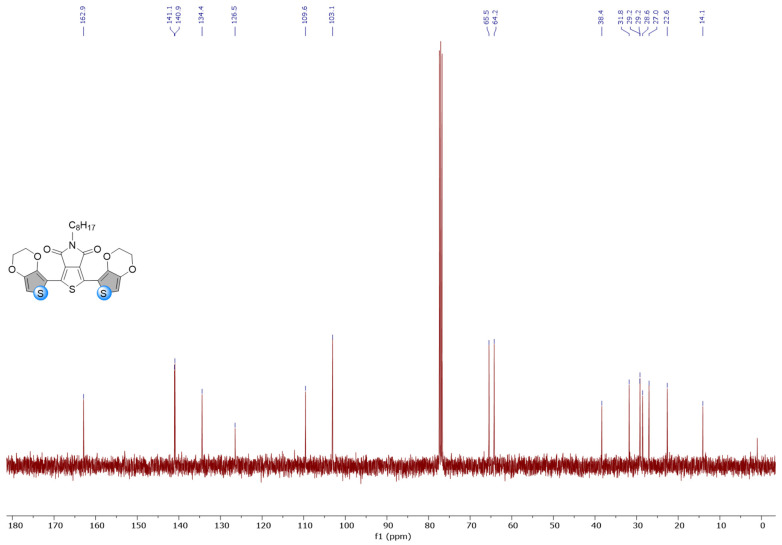
^1^H and ^13^C NMR spectra of **EDOT-TPD** in CDCl_3_.

**EDOT-TPD** (Figure A4). Yield 90%. Yellow crystals. ^1^H NMR (400 MHz, CDCl_3_): δ 6.52 (s, 2H), 4.45–4.24 (m, 8H), 3.60 (d, *J* = 7.4 Hz, 2H), 1.64 (q, *J* = 6.3 Hz, 2H), 1.34–1.21 (m, 10H), 0.85 (t, *J* = 6.7 Hz, 3H); ^13^C NMR (100 MHz, CDCl_3_): δ 162.9, 141.1, 140.9, 134.4, 126.5, 109.6, 103.1, 65.5, 64.2, 38.4, 31.8, 29.2, 29.2, 28.6, 27.0, 22.6, 14.1.

**Figure A5:**
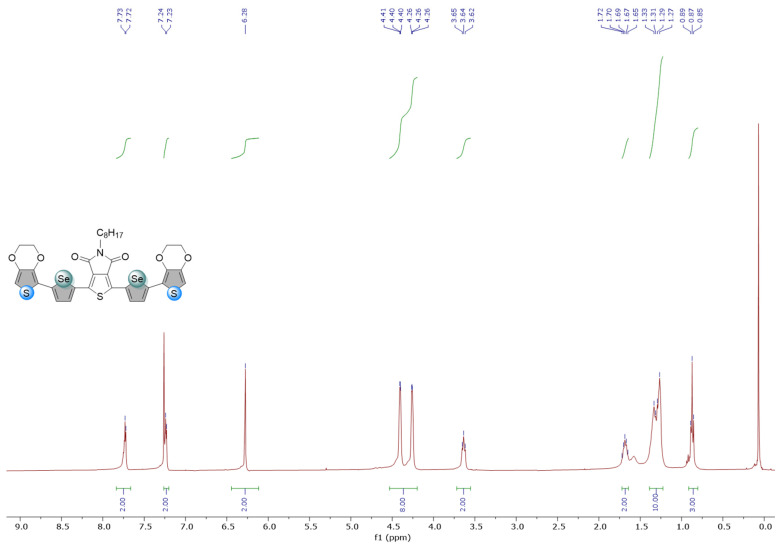

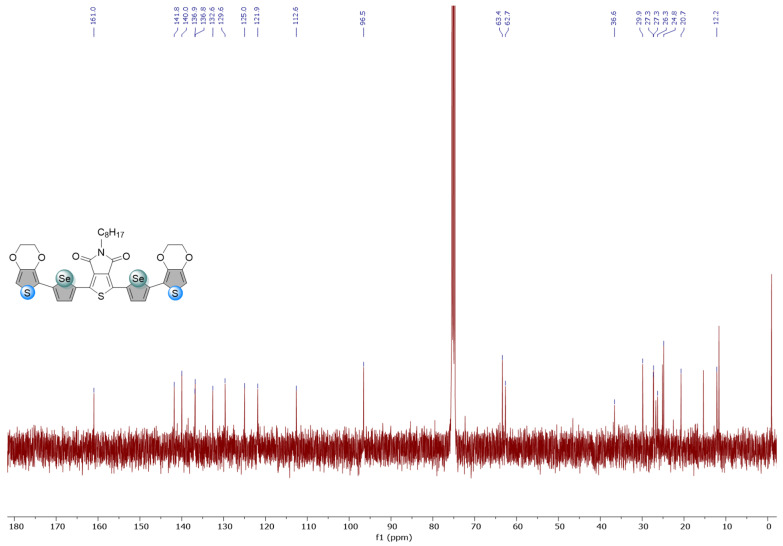
^1^H and ^13^C NMR spectra of **Se-EDOT-TPD** in CDCl_3_.

**EDOT-Se-TPD** (Figure A5). Yield 90%. Yellow crystals. ^1^H NMR (400 MHz, CDCl_3_): δ 7 7.73 (d, *J* = 4.0 Hz, 2H), 7.23 (d, *J* = 4.1 Hz, 2H), 6.28 (s, 2H), 4.53–4.20 (m, 8H), 3.63 (t, *J* = 7.2 Hz, 2H), 1.68 (q, *J* = 7.7 Hz, 2H), 1.39–1.23 (m, 10H), 0.87 (t, *J* = 6.8 Hz, 3H); ^13^C NMR (100 MHz, CDCl_3_): δ 161.0, 141.8, 140.0, 136.9, 136.8, 132.6, 129.6, 125.0, 121.8, 112.6, 96.5, 63.4, 62.7, 36.6, 29.9, 27.3, 27.3, 26.3, 24.8, 20.7, 12.2.

### 2.3. Biosensing Studies

**EDOT-TPD** was electrochemically deposited on the cleaned working electrode via CV scan for 15 cycles from −0.3 V to 1.0 V at 100 mV s^−1^, and the modified electrodes were washed with distilled water to remove contamination. After that, GOx enzyme (1.2 mg in 10 µL 50 mM pH 7.0 buffer) was dropped over a polymer-coated electrode surface, the GA crosslinking agent was used to modify the biomolecule-coated surface carefully for fixation (5.0 µL 1.0% in pH 7.0 50 mM phosphate buffer), and the GE/***poly*(EDOT-TPD**)/GOx biosensor was dried at room temperature for about 2 h. It was kept in the refrigerator (+4 °C) until it was used and it was washed well with distilled water before use. The performance of the GE/***poly*(EDOT-TPD**)/GOx biosensors for glucose was analyzed using chronoamperometric methods. The measurements were performed in a 10 mL 50 mM pH 7.0 phosphate buffer under mild stirring at −700 mV constant potential. A three-electrode system was constructed with a graphite rod electrode (WE), a platinum wire electrode (CE), and a pseudo-silver wire reference electrode (RE). The steady-state amperometric currents obtained before and after the addition of glucose were evaluated as a biosensor response.

## 3. Results

### 3.1. Synthetic Studies

**Figure 1:**
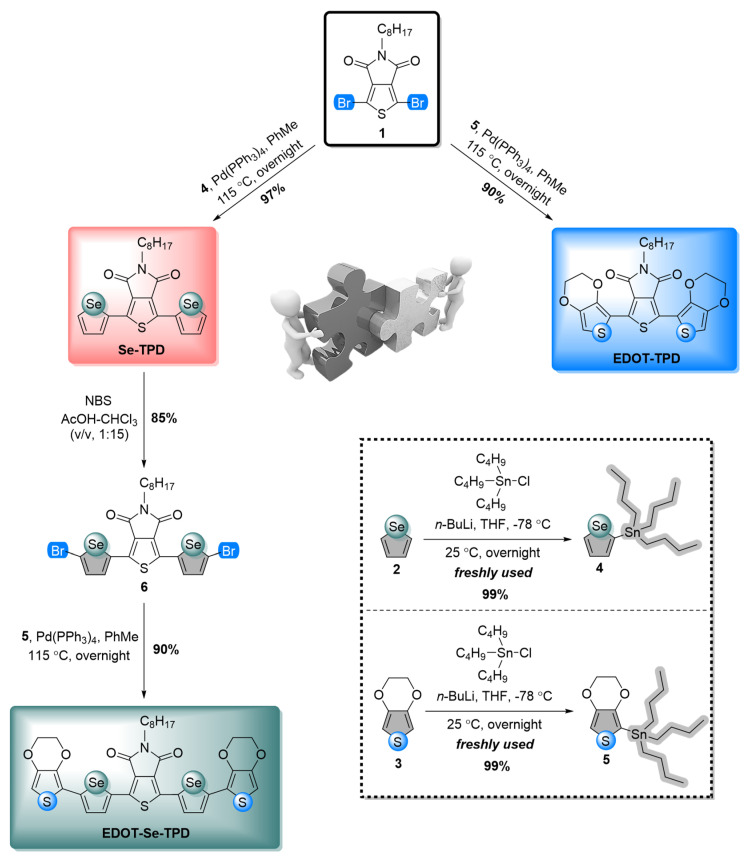
Practical synthetic pathways for the syntheses of selenophene- and EDOT-containing TPD-modified frameworks.

Three novel conjugated small organic materials with a thieno[3,4-*c*]pyrrole-4,6-dione (TPD) acceptor core, an *n*-octyl-based linear alkyl chain for solubility, and electron-rich heterocycles as the donors were synthesized via Pd-catalyzed Stille cross-coupling reactions. Pristine selenophene (molecule **2**) and EDOT (molecule **3**) moieties were initially mono-stannylated from their two positions and used for the next step without further purification. Then, these building blocks (molecules **4-5**) were separately coupled with molecule **1**, and target selenophene-containing (**Se-TPD**) and EDOT-containing (**EDOT-TPD**) novel monomers were achieved with high yields. To synthesize the EDOT extended derivative of **Se-TPD** (**EDOT-Se-TPD**), further facile two-step reactions were performed. In the first step, **Se-TPD** was bis-brominated with the treatment of NBS. In the final step, the previously synthesized mono-stannylated EDOT moiety (molecule **5**) was coupled with molecule **6** through Stille cross-coupling. The synthetic route of target monomers is given in Figure 1.

#### 3.1.1. Multi-Scan Cyclic Voltammetry Polymerization Studies

**Figure 2:**
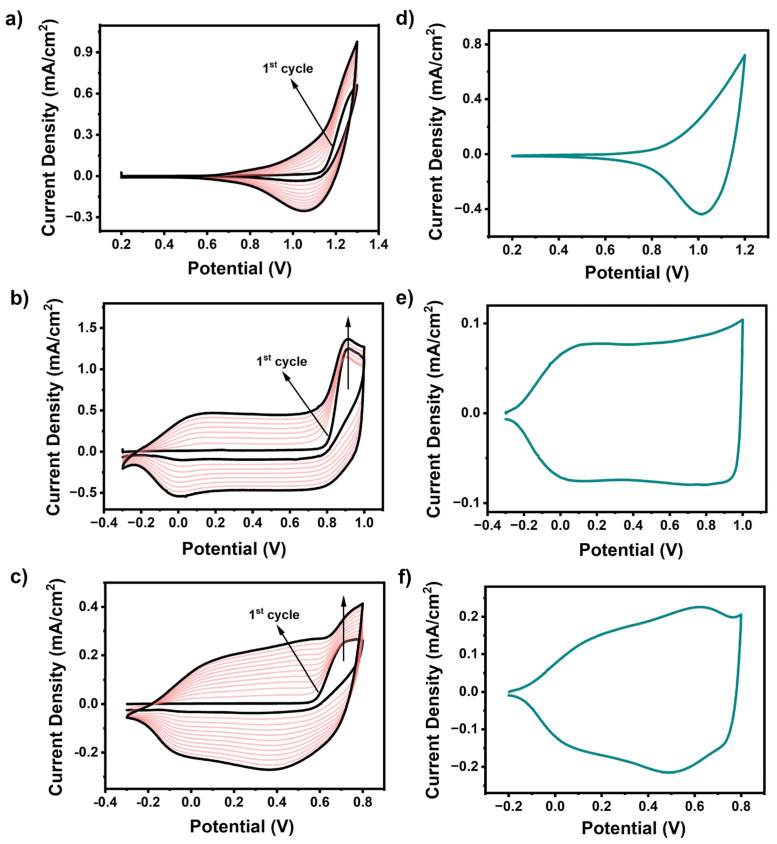
Multiple-scan cyclic voltammograms for electropolymerization of (**a**) **Se-TPD**; (**b**) **EDOT-TPD**; and (**c**) **EDOT-Se-TPD** at 100 mV.s^−1^ in 0.1 M PC/NaClO_4_-LiClO_4_-containing solvent/supporting electrolyte solution on ITO-coated glass slide; single-scan cyclic voltammograms of (**d**) **Se-TPD**; (**e**) **EDOT-TPD**; and (**f**) **EDOT-Se-TPD** in a monomer-free 0.1 M TBAPF_6_/ACN electrolyte solution on ITO-coated glass slide.

Each monomer except **Se-TPD** revealed an irreversible oxidation peak at +0.91 V (**EDOT-TPD**) and +0.72 V (**EDOT-Se-TPD**), respectively, with decreasing intensity in consecutive cycles (Figure 2). Although, no clear oxidation peak was identified for the **Se-TPD** monomer coloration of the polymerization medium, indicating the formation of soluble oligomers, and upon an extended number of scans, the thickness of the ***poly*(Se-TPD)** film gradually increased.

#### 3.1.2. Single-Scan Cyclic Voltammetry Studies

To investigate the redox behavior of the electrochemically produced polymers, single-scan cyclic voltammetry was performed in a monomer-free 0.1 M NaClO_4_/LiClO_4_/PC electrolyte solution at a scan rate of 100 mV·s^−1^ (Figure 2). The reversible *p*-doping/*p*-dedoping peaks of the corresponding polymer films of ***poly*(EDOT-TPD)** and ***poly*(EDOT-Se-TPD)** were detected at +0.073 V/+0.14 V and +0.62 V/+0.52 V, respectively. Although the *p*-dedoping peak of ***poly*(Se-TPD)** was located at +1.02 V, the corresponding *p*-doping peak was not clearly observed. Due to their electron-rich nature, the electrochemical polymerization of **Se-TPD**, **EDOT-TPD**, and **EDOT-Se-TPD** resulted in *p*-type semiconducting materials, and hence did not show *n*-type behavior in cyclic voltammogram (CV) studies. Typically, for these types of materials, only HOMO energy levels could be calculated from the onset of the oxidation potentials of their *p*-doped state ($E_{ox}^{onset}$). **Se-TPD** showed apparent oxidation potential, and therefore the oxidation potential onset of this novel monomer could be detected at +0.90 V in the anodic region. Then, the corresponding HOMO energy level was calculated as −5.65 eV using the standard formula ($HOMO=-\left(4.75+E_{ox}^{onset}\right)eV$). Since there was not any obvious oxidation potential onset in the anodic scan for both **EDOT-TPD** and **EDOT-Se-TPD** due to their capacitive characteristics, their corresponding HOMO energy levels could not be calculated from CV studies.

### 3.2. Spectroelectrochemical Studies

#### 3.2.1. Optical Studies

**Figure 3:**
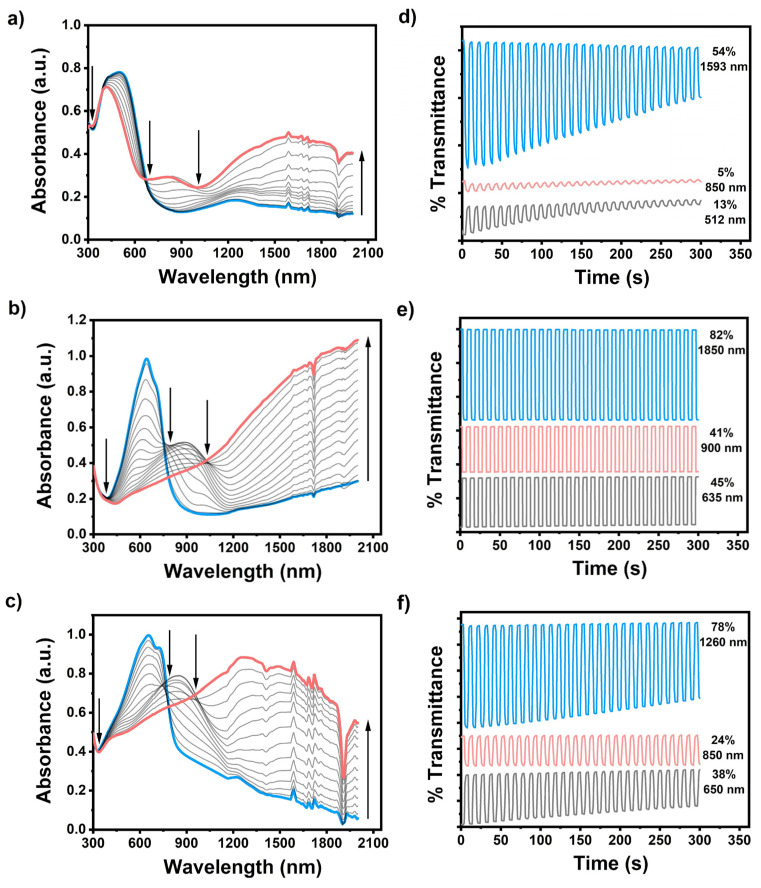
Electronic absorption spectra of (**a**) **Se-TPD**; (**b**) **EDOT-TPD**; and (**c**) **EDOT-Se-TPD** upon *p*-doping, ranging between 0.0 V and +1.3 V, +1.0 V, and +0.8 V, respectively, in a monomer-free 0.1 M TBAPF_6_/ACN electrolyte solution on ITO-coated glass slide; the optical contrasts of (**d**) **Se-TPD**; (**e**) **EDOT-TPD**; and (**f**) **EDOT-Se-TPD** in a monomer-free 0.1 M TBAPF_6_/ACN electrolyte solution on ITO-coated glass slide at their maximum absorption points.

Based on the optical studies, several crucial potential-dependent parameters, covering the maximum absorption ($\lambda_{max}$), the maximum absorption onset ($\lambda_{max}^{onset}$), the optical band gap ($E_{g}^{op}$), and the polaron and bipolaron bands of the conjugated polymer films, were distinctly identified (Figure 3). The neutral conjugated polymer films **Se-TPD**, **EDOT-TPD**, and **EDOT-Se-TPD** displayed maximum absorbance at 512 nm, 635 nm, and 650 nm, respectively, which was associated with the inter band π-π* transitions of the neutral forms of these polymeric films. The corresponding onset values were estimated at 733 nm, 807 nm, and 875 nm, respectively. These above-mentioned results indicated that TPD-modified polymer films with EDOT building blocks (***poly*(EDOT-TPD)**) displayed broader and more red-shifted absorption compared with their selenophene-containing counterpart (***poly*(SE-TPD)**), which is consistent with the literature results. On the other hand, ***poly*(EDOT-Se-TPD)** showed a much broader absorption band than polymer films containing only one of the donor building blocks.

When the applied potential gradually increased, each polymer film started to oxidize, and therefore new absorption points, caused by radical-based cations (polaron) and dications (bipolarons), became dominant. The optical band gaps of the resultant polymer films, specified as the onset of the π-π* transitions, were separately identified as 1.69 eV, 1.54 eV, and 1.42 eV using the equation $E_{g}^{op}=1241/\lambda_{max}^{onset}$) for **Se-TPD**, **EDOT-TPD**, and **EDOT-Se-TPD, respectively**.

#### 3.2.2. Kinetic Studies

The optical contrasts for electrochemically produced ***poly*(Se-TPD), *poly*(EDOT-TPD)**, and ***poly*(EDOT-Se-TPD)** were determined as a percentage transmittance change (ΔT%) at $\lambda_{max}$ (Figure 3). Among them, ***poly*(Se-TPD)** revealed a low optical contrast change in both the visible (13% at 512 nm) and NIR region (5% at 850 nm). Its optical contrast in the bipolaronic regions was higher, as expected (54% at 1593 nm). On the other hand, both **poly(EDOT-TPD)** and ***poly*(EDOT-Se-TPD)** displayed relatively high optical contrasts in visible (45% and 38%, respectively) and outstanding optical contrasts in NIR regions (82% for ***poly*(EDOT-TPD)** at 1850 nm and 78% for ***poly*(EDOT-Se-TPD)** at 1260 nm).

The switching times for the polymers were estimated to be at 95% of the full switch at the different wavelengths. The switching times of ***poly*(Se-TPD)**, ***poly*(EDOT-TPD)**, and ***poly*(EDOT-Se-TPD)** are summarized in Table 1. In general, the switching times of the TPD-based polymers were relatively long compared to the donor–acceptor-type materials that utilize similar donors but different acceptors [56,57,58,59,60,61].

In addition to the fast switching times and high optical contrasts, the multi-chromic behavior is highly sought after for several electrochromic device applications. Table 2 represents the color coordinates of the conjugated copolymer films of ***poly*(Se-TPD)**, ***poly*(EDOT-TPD)**, and ***poly*(EDOT-Se-TPD)** in the Commission internationale de l’éclairage (CIE) L*a*b* color space and their corresponding photographs in neutral and oxidation states. All three materials showed three distinct colors in neutral, partially oxidized, and fully oxidized states.

### 3.3. Biosensor Studies

#### 3.3.1. Optimization Studies and Investigation of Electrochemical and Surface Characteristics of the Biosensor

**Figure 4:**
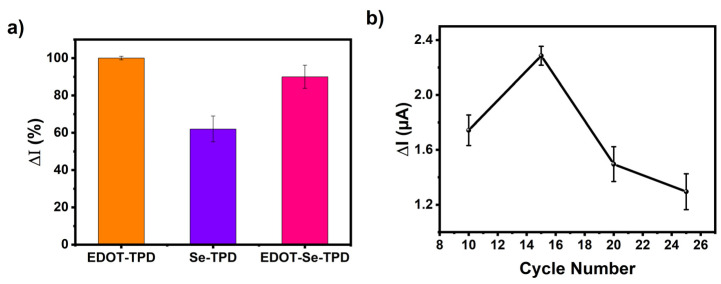

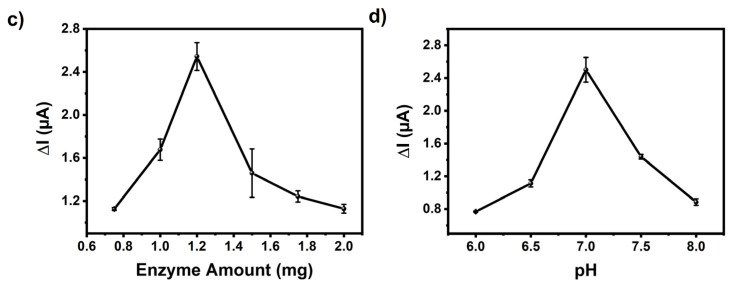
(**a**) Performance evaluation of the biosensors (GE/***poly*(EDOT-TPD)**/GOx; GE/***poly*(Se-TPD)**/GOx; and GE/***poly*(EDOT-Se-TPD)**/GOx) and optimization of (**b**) cycle number, (**c**) amount of GOx, and (**d**) pH on the biosensor response.

To evaluate the sensor performances of the TPD-based conjugated polymers, three different electrodes were constructed for glucose sensing (**GE/*poly*(EDOT-TPD)**/GOx; GE/***poly*(Se-TPD)**/GOx; and GE/***poly*(EDOT-Se-TPD)**/GOx). When the electrode was fabricated with ***poly*(EDOT-TPD)**, the amperometric current was at its highest (Figure 4a). The related surface showed favorable sensing abilities toward glucose, with its fast response, good film-forming ability, and remarkable electrochemical properties. Although the selenium-bearing polymers provided remarkable improvements in the biosensor studies [62], the performance of the materials utilized in this work was lacking compared to ***poly*(EDOT-TPD)**. Slow responses and unstable signals were observed, which were attributed to improper localization of the biomolecules on the electrode surface, especially for the ***poly*(Se-TPD)**-based sensors. The biosensor based on ***poly*(EDOT-Se-TPD)** revealed around 10% decreased performance in the current response compared to the biosensors based on ***poly*(EDOT-TPD)**. As a result, to create stable sensing frameworks for glucose, GE/***poly*(EDOT-TPD)**/GOx was selected after initial screening and further optimization studies were performed.

The influence of cycle number, enzyme amount, and pH on the electrochemical sensing performance was investigated in detail. First of all, the cycle number was considered since a thick film on the transducer can hamper electron transfer, resulting in a longer response time. On the other hand, a thinner film causes an improper interaction of the biomolecules with the polymer. The polymerization was performed between 10 and 25 cycles and their responses were compared. The GE/***poly*(EDOT-TPD)**/GOx biosensor produced by 15 cycles, which corresponds to 125 nm (equivalent to 80.8 mC charge) in thickness, was found to be optimal (Figure 4b). Later, sensors with different enzyme amounts were fabricated with 0.75, 1.0, 1.2, 1.5, 1.75, and 2.0 mg GOx in phosphate buffer (50 mM pH 7.0) (Figure 4c). The optimum enzyme amount was found to be 1.2 mg GOx in the GE/***poly*(EDOT-TPD)**/GOx biosensors. Finally, the pH optimizations were performed. According to the literature, GOx catalytic activity can be determined in a wide range from pH 6.0 to 8.0 [63]. Here, the pHs in this range with 0.5 increments were investigated (Figure 4d) and it was found that the GE/***poly*(EDOT-TPD)**/GOx biosensor showed the highest responses at pH 7.0.

**Figure 5:**
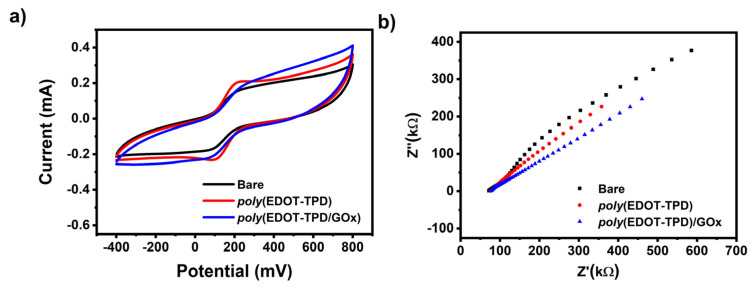
(**a**) CV and (**b**) EIS of a bare GE, GE/***poly*(EDOT-TPD)**, and GE/***poly*(EDOT-TPD(C)**/GOx in 50 mM pH 7.0 buffer containing 5.0 mM [Fe(CN)6]^3−/4−^ and 0.1 M KCl.

Cyclic voltammetry (CV) and electrochemical impedance spectroscopy (EIS) were performed to verify the stepwise electrode modification process. Figure 5a shows the CV responses of different modified electrodes in a 0.5 mM [Fe(CN)_6_]^3−/4−^ solution containing 0.1 M KCl at a scan rate of 50 mV/s. Clearly, the electrochemical response of GE/***poly*(EDOT-TPD)** was greatly improved when the ***poly*(EDOT-TPD)** structures were grown on the GE surface. The high surface area and good conductivity of ***poly*(EDOT-TPD)** should be credited for this improved cyclic voltammetric response. The effective surface areas of the modified electrodes were estimated using the Randles–Sevcik equation [64] as 0.26 cm^2^ and 0.2 cm^2^ for GE/***poly*(EDOT-TPD)** and GE/***poly*(EDOT-TPD)**/GOx, respectively.

Another effective method for analyzing the interface characteristics of GE with polymer and GOx film modification is electrochemical impedance spectroscopy (EIS). EIS experiments were performed in a 5.0 mM [Fe(CN)_6_]^3−/4−^ solution, including 0.1 M KCl. Figure 5b displays the impedance spectra of bare GE, GE/***poly*(EDOT-TPD)**, and GE/***poly*(EDOT-TPD)**/GOx. The introduction of ***poly*(EDOT-TPD)** served an outstanding film-forming ability and encouraged the electron transport of [Fe(CN)_6_]^3−/4−^ to the surface of the electrode, which may be responsible for the improvement in the interfacial conductivity. After the GOx was combined with ***poly*(EDOT-TPD)** to modify GE, the electron transfer resistance changed, indicating that the GOx had been successfully immobilized on the electrode surface. The CV and EIS results proved the success of the electrode modification and were in good agreement with each other.

**Figure 6:**
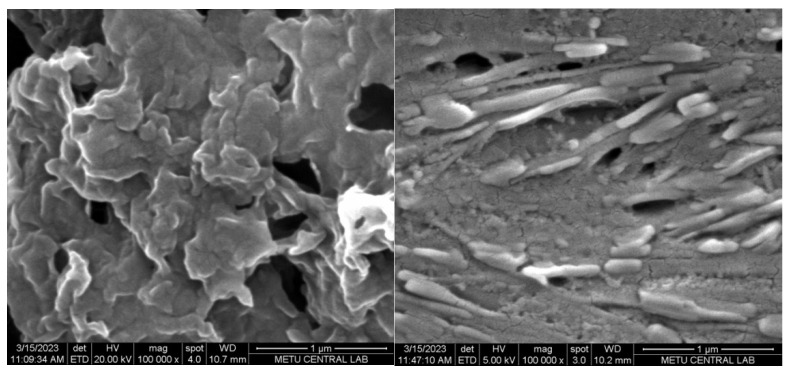
**FE**-SEM images of GE/***poly*(EDOT-TPD)** (**left**) and GE/***poly*(EDOT**-**TPD**)/GOx (**right**) under optimal conditions.

Field emission scanning electron microscopy (FE-SEM) images were used to analyze the surface morphology of the generated electrodes. A porous layer of the polymer was coated on the bare GE, as seen in Figure 6 (left), which shows the ***poly*(EDOT-TPD)**-coated GE. The ***poly*(EDOT-TPD)** layer’s porosity (typical cauliflower-type structures of polymer) makes it easier for the electrolyte to enter the electrode. After the surface was modified with GOx, enzyme immobilization was shown by the formation of a warped smooth surface over the porous layer, as seen in Figure 6 (right). The electrode’s specific surface area is improved by the enzyme’s uniform distribution.

#### 3.3.2. Analytical Characterization of the GE/poly(EDOT-TPD)/GOx Biosensor

**Figure 7:**
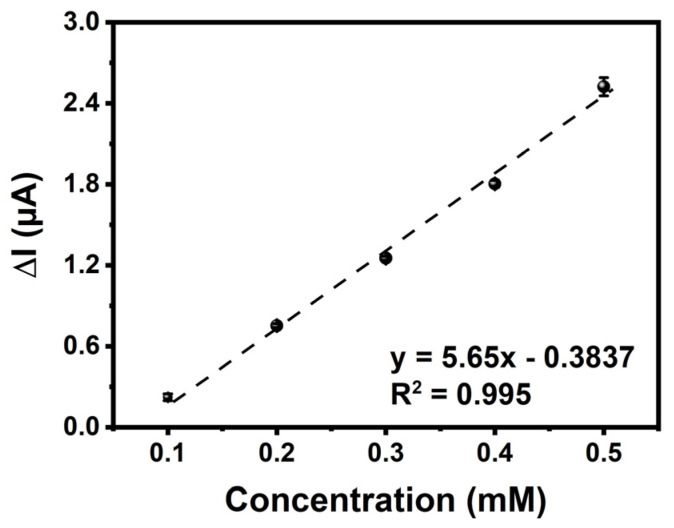
Amperometric current–time response curves of GE/***poly*(EDOT-TPD)**/GOx with successive additions of glucose of different concentrations into a stirred 50 mM phosphate buffer (pH 7.0, 10 mL). Applied potential: −700 mV versus pseudo-silver wire.

Specific steady-state current as a function of glucose concentration was demonstrated to have a linear relationship in the 0.1–0.5 mM range (y = 5.65x − 0.3837 (R^2^ = 0.995)) (Figure 7). The biosensor sensitivity was revealed by calculating the values of the limit of quantification (LOQ) and limit of detection (LOD), utilizing the associated equations [65,66]. The GE/***poly*(EDOT-TPD)**/GOx showed a LOD value of 0.018 mM and a LOQ value of 0.054 mM with a sensitivity of 65.765 μA mM^−1^cm^−2^. The sensitivity and LOD value of the biosensor are remarkably higher than those of other glucose biosensors reported (Table 3) in the literature. The high specific surface area of ***poly*(EDOT-TPD)**’s cauliflower-like structure allowed for an increase in the amount of enzyme surface loading, which effectively increased the active surface area of the GE that was available for enzyme immobilization. As a result of the ***poly*(EDOT-TPD)** enzyme structure, the enzyme was able to maintain its stability and function. For the sensitive and fast detection of glucose, thieno[3,4-*c*]pyrrole-4,6-dione (TPD)-centered polymer-based biosensors have the potential to be reliable and promising electrochemical biosensing platforms. In addition, the apparent Michaelis–Menten constant (Kmapp) and Imax values were estimated as being 0.608 mM and 1.17 μA, respectively, using the Lineweaver–Burk equation. The lower Km value signifies an enzymatic function and an increased binding affinity of the immobilized GOx to the GE/***poly*(EDOT-TPD)**.

**Figure 8:**
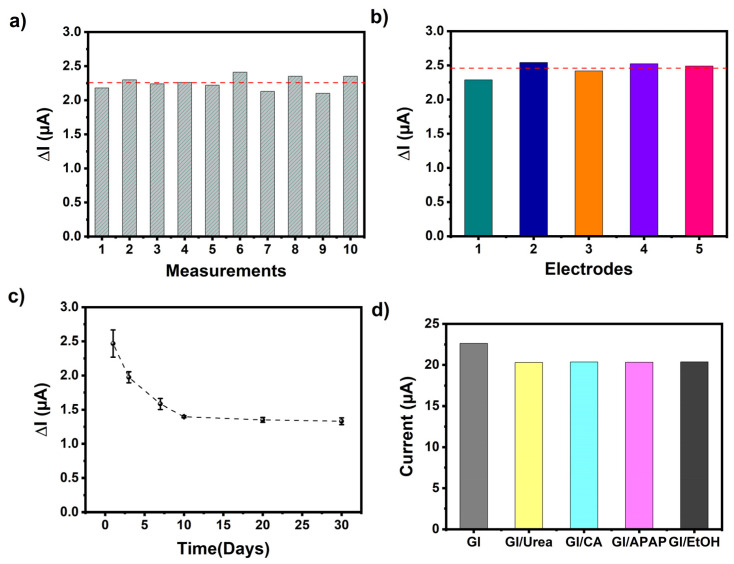
(**a**) Repeatability, (**b**) reproducibility, (**c**) shelf-life, and (**d**) interference results of the GE/***poly*(EDOT-TPD(C8)-EDOT)**/GOx biosensor (red dashed line indicates I_avarage_ of the results).

The performance of the designed biosensor was further characterized through reproducibility and repeatability tests and shelf-life experiments. Figure 8a shows the repeatability of the GE/***poly*(EDOT-TPD)**/GOx for ten successive amperometric signals toward 0.5 mM glucose. A low-percentage relative standard deviation (% RSD) was determined as being 4.4. To analyze the reproducibility, five new GE/***poly*(EDOT-TPD)**/GOx electrodes were prepared in the same conditions but on different days, and their amperometric responses were recorded. As shown in Figure 8b, no significant change was observed in the current signal of any electrode, and the % RSD of 4.2 displayed the high reproducibility of the designed biosensor. All of these results demonstrated the outstanding repeatability and reproducibility of the GE/***poly*(EDOT-TPD)**/GOx biosensor. For the long-term stability of the proposed biosensor, the amperometric responses of the newly prepared GE/***poly*(EDOT-TPD)**/GOx biosensor were tested for 30 days. According to the results, it maintained 54% of its initial current even after 30 days of storage at 4 °C, and a negligible decrease was observed later, which demonstrates remarkable long-term stability (Figure 8c).

Furthermore, one of the most critical factors for detecting the target analyte and differentiating between different interfering chemicals is the selectivity of the proposed system. The selectivity of the designed biosensor toward glucose could be affected by several other generally coexisting interfering chemicals, for example, ethanol (EtOH), acetaminophen (APAP), citric acid (CA), and urea. The amperometric responses followed for all interfering chemicals (0.25 mM) in a 50 mM pH 7.0 phosphate buffer at −700 mV were insignificant compared to the clear and fast current signal for glucose (Figure 8d). These findings indicate that the designed biosensor exhibits excellent selectivity and can be applied for practical use.

#### 3.3.3. Real Sample Analysis of GE/poly(EDOT-TPD)/GOx Biosensor

By incorporating spiked glucose into the Coke^®^ Zero sugar sample, the recovery test was performed in order to assess the effectiveness of the GE/***poly*(EDOT-TPD)**/GOx biosensor for real sample analysis. When the Coke^®^ Zero sugar sample was tested with the biosensor, no amperometric response was recorded, as expected. Once the drink sample was spiked with different concentrations of glucose (0.3 and 0.5 mM), significant amperometric responses were observed (Table 4). The samples were tested using amperometry for determining the response of the beverage spiked with 0.3 mM and 0.5 mM glucose, and the % recovery values were estimated as being 100.5 and 100.2, respectively. The outcomes show that the biosensor in its as-fabricated state is a directly usable sensing platform for the quick and accurate detection of glucose in an actual beverage sample.

## 4. Conclusions

In this study, a family of three conjugated monomers (**Se-TPD**, **EDOT-TPD**, and **EDOT-Se-TPD**), based on electron-deficient thieno[3,4-*c*]pyrrole-4,6-dione (TPD) as the acceptor and two common electron-rich building blocks (selenophene and EDOT) as the donor, was synthesized via Pd-catalyzed Stille cross-coupling and electrochemically polymerized. Among the three materials, ***poly*(EDOT-TPD)** demonstrated the best characteristics for electrochromic device applications, considering optical contrast and switching time values. Additionally, ***poly*(EDOT-TPD)** was also shown to be the best candidate for the fabrication of glucose biosensing platforms. The sensitivity and LOD value of the biosensor are remarkably higher than those of other glucose biosensors reported in the literature, and the utilization of the biosensor electrodes in real-life samples was also successfully demonstrated. While other selenophene-containing donor–acceptor polymers showed high potential for electrochromic device applications and biosensors, with the TPD acceptor, selenophene was shown to have detrimental effects compared to its EDOT counterpart. This study clearly demonstrates that the performance of donor–acceptor polymers in electrochromic and biosensing applications heavily depends on the unique combination of the donor and acceptor moieties, and should shine light on further design efforts for improved performance in corresponding applications.

## Figures and Tables

**Table 1:** Summary of optical and kinetic studies of innovative TPD-based conjugated monomers, modified with selenophene and EDOT scaffolds.

	Optical Studies				Kinetic Studies	
Monomer	$\lambda_{max}$ (nm)	$\lambda_{max}^{onset}$ (nm)	$E_{g}^{op}$ (eV)	$E_{LUMO}$ (eV)	ΔT (%)	$t_{switching}$ (s)
1 *	512	733	1.69	−3.96	13 (512 nm) 5 (850 nm) 54 (1593 mm)	5.1 (512 nm) 6.3 (850 nm) 6.0 (1593 mm)
2 *	635	807	1.54	-	45 (635 nm) 41 (900 nm) 82 (1850 mm)	5.2 (635 nm) 5.1(900 nm) 5.1 (1850 mm)
3 *	650	875	1.42	-	38 (650 nm) 24 (850 nm) 78 (1260 mm)	6.1 (650 nm) 5.2 (850 nm) 5.3 (1260 mm)

1 * Se-TPD; 2 * EDOT-TPD; 3 * EDOT-Se-TPD.

**Table 2:** L*a*b* color coordinates of (**a**) ***poly*(Se-TPD)**; (**b**) ***poly*(EDOT-TPD)**; and (**c**) ***poly*(EDOT-Se-TPD)** polymer thin films in their neutral and fully oxidized states.

*poly*(Se-TPD)			*poly*(EDOT-TPD)			*poly*(EDOT-Se-TPD)		
+0.2 V		+1.2 V	−0.3 V		+1.0 V	−0.3 V		+0.8 V
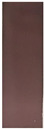	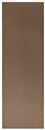	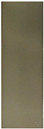	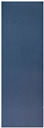	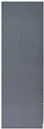	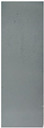	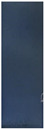	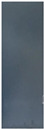	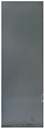
**L**: 32.24	**L**: 42.93	**L**: 44.02	**L**: 33.00	**L**: 45.68	**L**: 5515	**L**: 26.40	**L**: 37.97	**L**: 41.43
**a**: 13.12	**a**: 6.773	**a**: −0.905	**a**: −3.054	**a**: −1.085	**a**: −3.577	**a**: −2.054	**a**: −2.624	**a**: −2.413
**b**: 6.648	**b**: 15.53	**b**: 15.62	**b**: −19.33	**b**: −6.546	**b**: −0.663	**b**: −13.58	**b**: −7.288	**b**: −0.851

**Table 3:** Comparison of the analytical performances of glucose biosensors.

Modified Electrode	Linear Range [mM]	LOD [mM]	Sensitivity [µA/(mM·cm^2^)]	Application	Ref.
GCE/MWCNTs-RuO_2_/GOx/Nafion	0.1–0.8	17.4 × 10^−3^	NA	Heavy metals	[67]
VACNF/HRP/GOx	4.0 × 10^−4^–4.0 × 10^−2^	4.0 × 10^−4^	89.035	NA	[68]
P(EDOT-PdBPI-co-HKCN)/GOx	0.25–2.5	0.176	NA	Coke Juice	[69]
GOx-TiCNFs	0.013–10.5	3.7 × 10^−3^	628.82	Human serum	[70]
CS/GOx/MnO_2_-CNFs	0.08–4.6	0.015	1.425	Human urine	[71]
GOx/Gold/MoS_2_/ Gold	0.5–10.0	0.01	NA	Human serum	[72]
GOx/GA/GN/GCE	0.5–90	0.06	NA	Beverages	[73]
PET/CNF/P-BDT-BTz:BDA/GOx	0.02–0.5	8.5 × 10^−3^	98.192	Coke^®^Zero Sugar and CapriSun^®^	[74]
CTS/Nafion/GS/GOx	8.17 × 10^−3^–1.0	2.45 × 10^−3^	1.790	Human sweat	[75]
P(ProTThia)/CHIT/ MWCNT/GOx	0.01–0.75	3.2 × 10^−2^	63.76	L^®^Ice tea	[76]
**GE/*poly*(EDOT-TPD)/GOx**	**0.1–0.5**	**0.018**	**65.765**	**Coke^®^Zero Sugar**	**This work**

**Table 4:** Glucose analysis in drink with the GE/***poly*(EDOT-TPD)**/GOx.

Sample			
Coke^®^Zero Sugar	Spiked with Glucose (mmol/L)	Found with the Biosensor (mmol/L)	Recovery (%)
	0.0	0.07 ± 0.0031	–
	0.30	0.301 ± 0.0061	100.3
	0.50	0.501 ± 0.0054	100.2

## Data Availability

Not applicable.

## Appendix A

The structural analyses of all synthesized materials were identified via ^1^NMR and ^13^C NMR studies to show their purity, which are shown as follows.
